# Supplementary material for: Animal invaders threaten protected areas worldwide
Source: Nat Commun. 2020 Jun 8;11:2892. doi: 10.1038/s41467-020-16719-2 (PMC7280267; doi:10.1038/s41467-020-16719-2)
Supplement: Supplementary file 1 — Supplementary Information [file 41467_2020_16719_MOESM1_ESM.pdf]

Supplementary Information

Title: Alien animals threaten protected areas worldwide

Liu et al.

Supplementary Fig. 1

A flow chart illustrating the process of data collection, screening and analyses to determine the alien animal richness, correlates and potential risks in protected areas at the global scale.

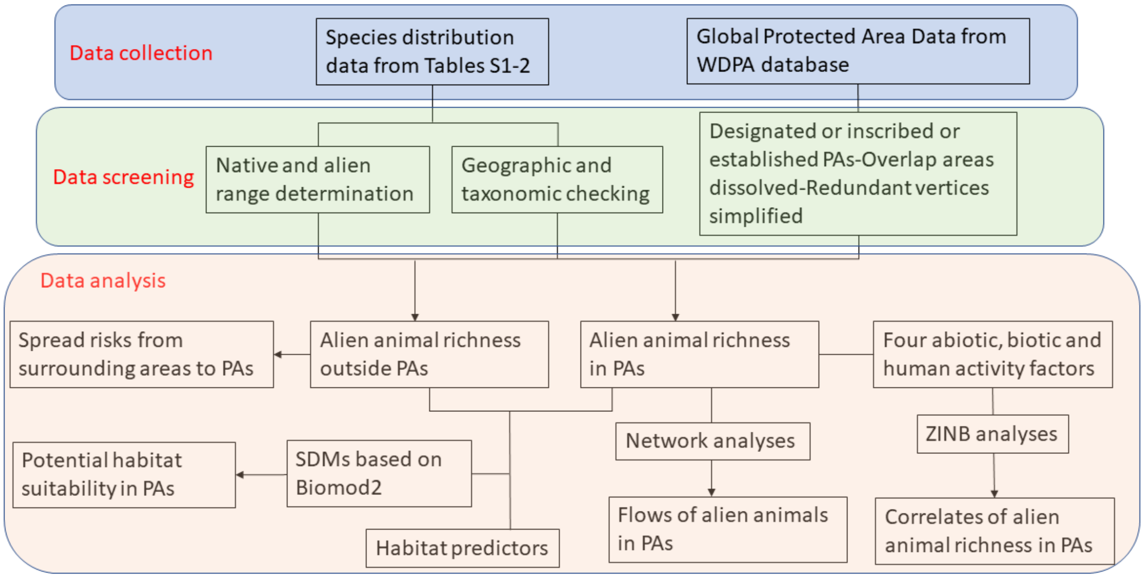

Supplementary Fig. 2

Variations in spatial distributions of 894 established alien animals in 199,957 global terrestrial protected areas across taxa. Credit: Tianjian Song (animal silhouettes).

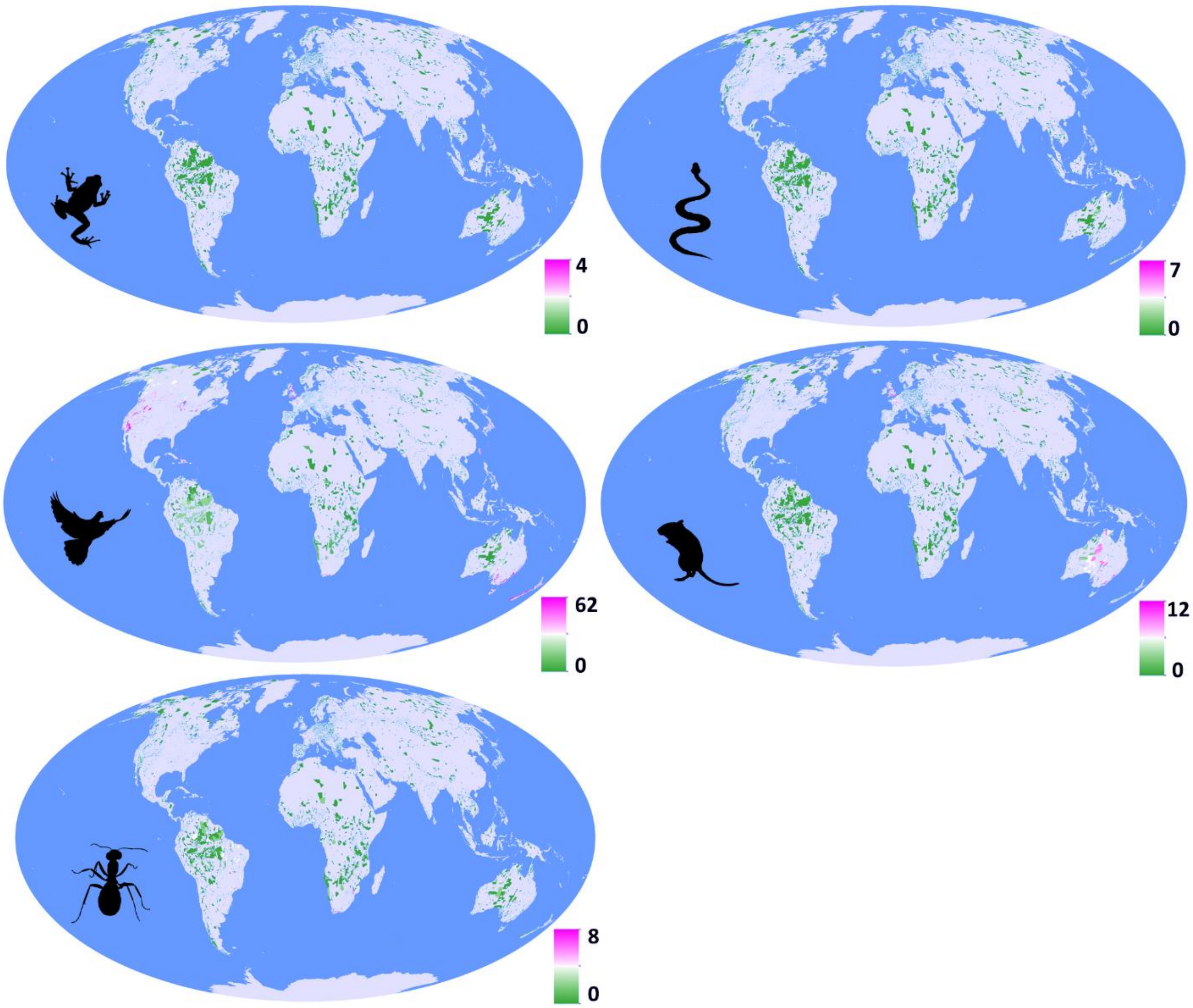

Supplementary Fig. 3

Richness of established alien animal in island and mainland PAs. The insular PAs were defined as those the boundary of which was completely located on an island. Values are shown as mean ±s.e.m.

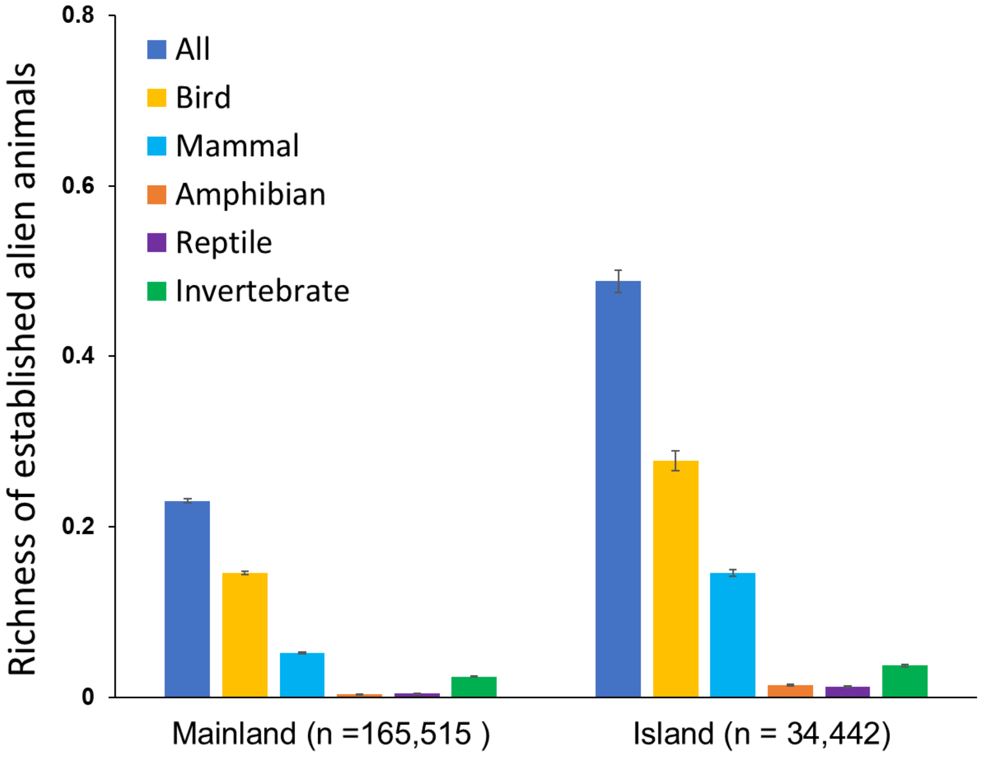

Supplementary Fig. 4

Richness of established alien animals across 14 global ecoregions (biomes) for each taxonomic group among IUCN onservation categories. Values are shown as mean ±s.e.m., the number of PAs with different conservation categories in each biome were provided in brackets.

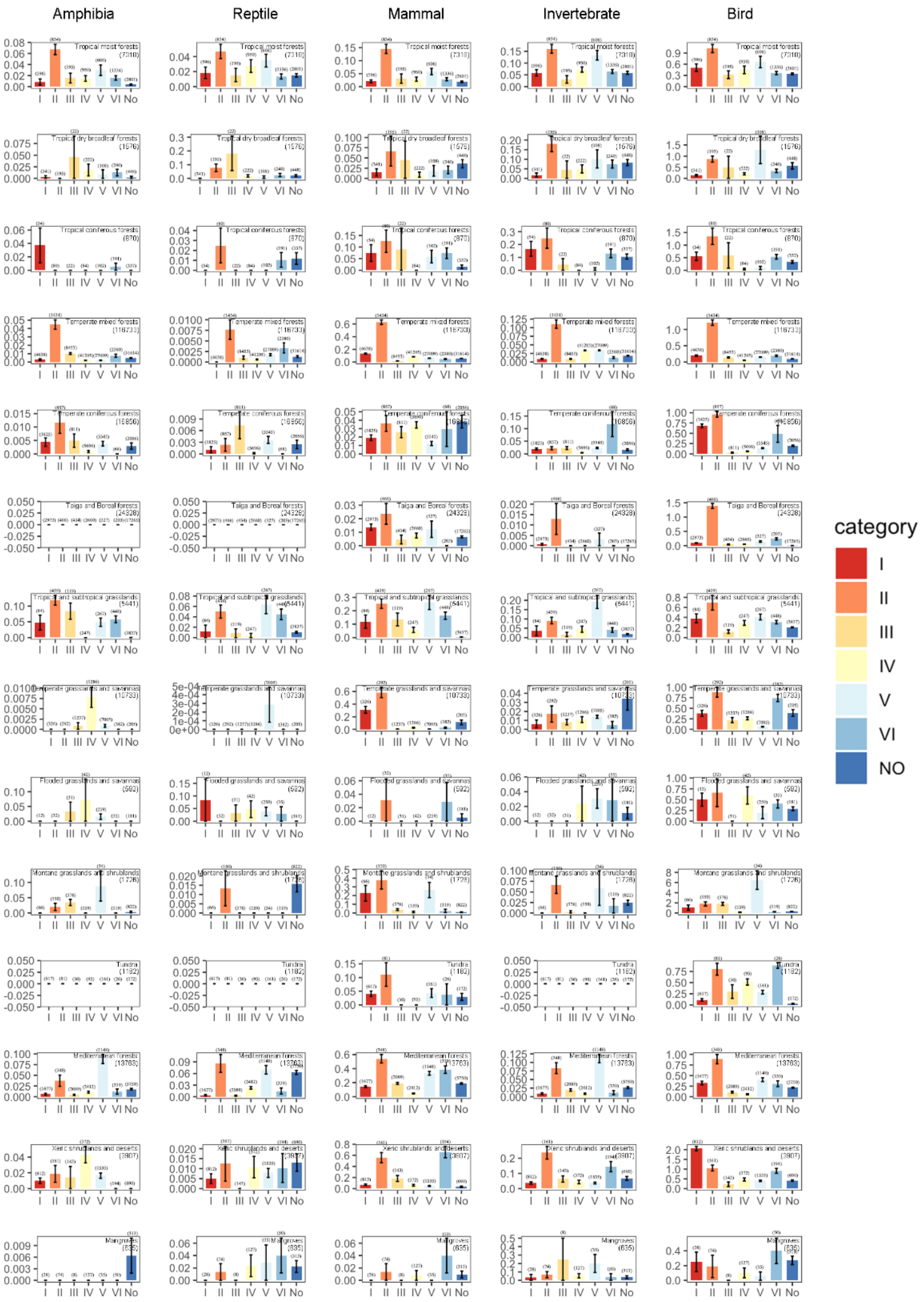

Supplementary Fig. 5

Fixed-effect parameter estimates of surface area, designation year, native species richness and human footprint on richness of established alien animal species across taxonomic groups at finer resolution (5 km × 5 km) and coarser resolution (20 km × 20km). Estimates of effects were obtained from zero-inflated negative binomial mixed-effects models with four abiotic, biotic and human activity factors as fixed effects and country identity as a nested random effect. Note that native species richness was used only in the model for vertebrate groups due to the unavailability of invertebrate native distribution data. A constant of 1 was added to each variable prior to log transformation. Credit: Tianjian Song (animal silhouettes).

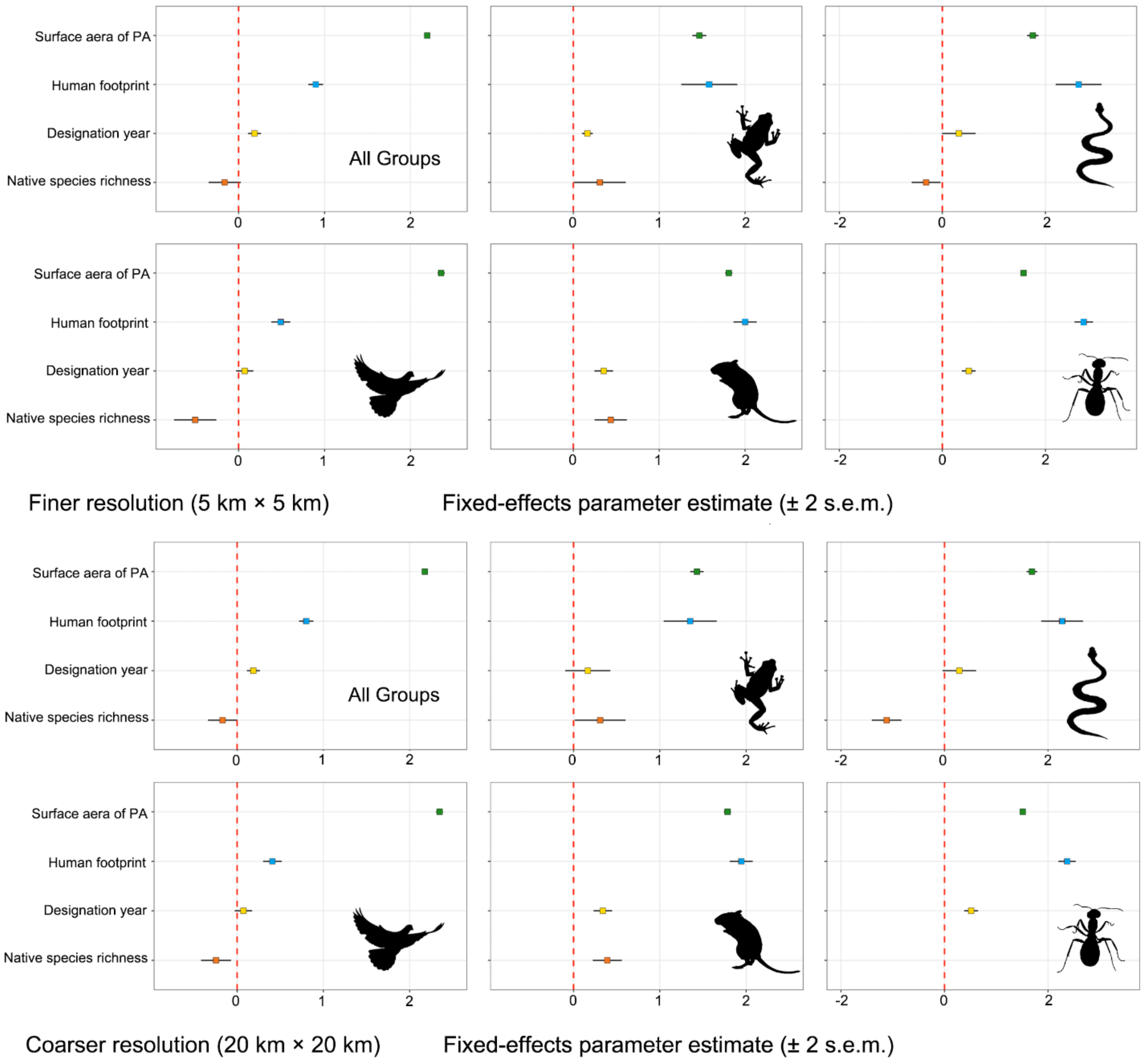

Supplementary Fig. 6

Comparisions of four abiotic, biotic and anthropogenic predictor variables of established alien animal richness in PAs among IUCN conservation categories. Values are shown as mean ± s.e.m.

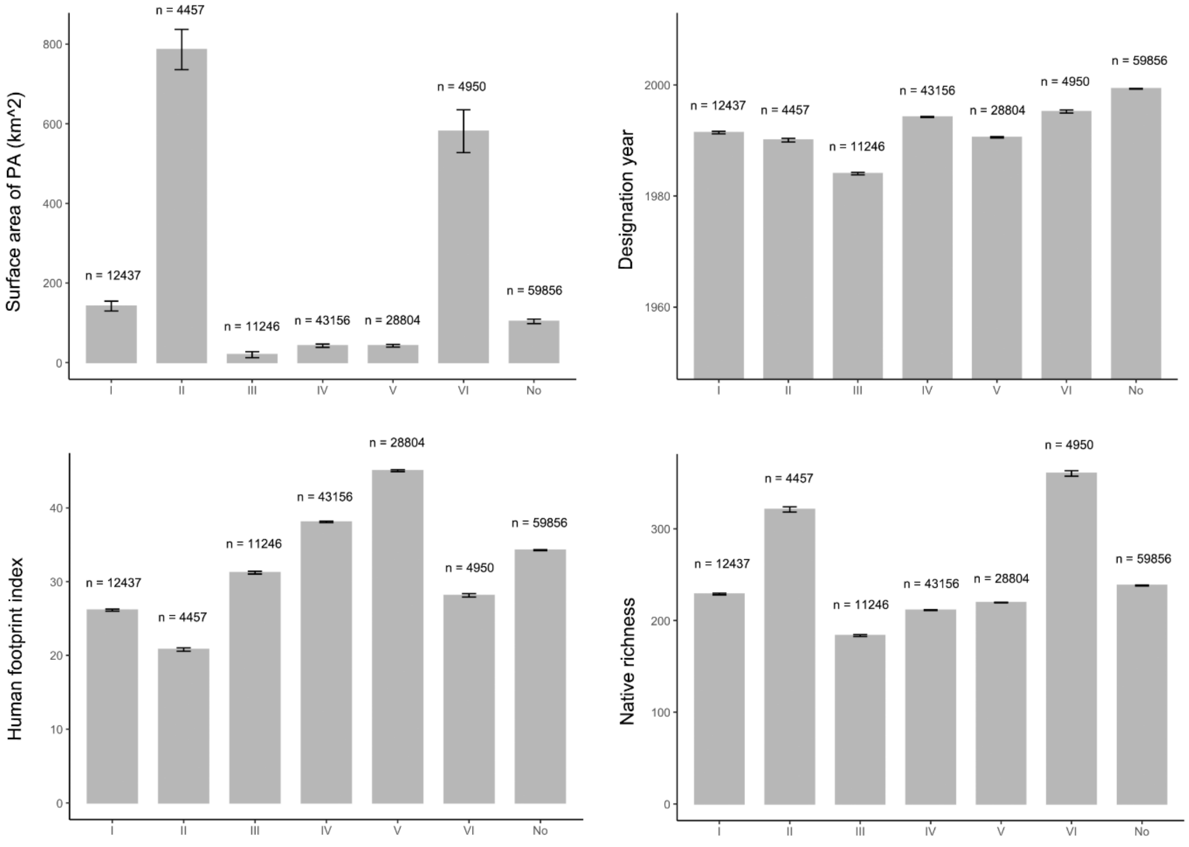

Supplementary Table 1

Overview of the process of data collection, screening and analysis (the whole flow is shown in Supplementary Fig. 1).

Stage	Content	Description	Software
Data collection	Species distribution data	We collected occurrence data of 894 alien animals from 16 different public databases and an intensive review of 959 published references (Supplementary Data 1-2).	Manual
	Global Protected Area Data	We obtained data on the location, boundary, designation year, and area of 199,957 global protected areas from the World Database on Protected Areas.	ArcGIS
Data screening	Native and alien range determination	We discriminate the exact native and invaded range information for each species based on IUCN maps, Kraus' (2009) compendium, Capinha et al (2017), Liu et al. (2019), BLINS, GAVIA, and Long's book (2009). References are shown in the main text.	Manual
	PAs dissolved and simplified	We followed WDPA best practice guidelines to dissolve the overlapped areas into a single polygon and applied the "simplify" function in ArcGIS to remove redundant vertices of PA polygons.	ArcGIS
Data analysis	Flows of alien animals in PAs	Supplementary Method 1	R
	Correlates of alien richness in PAs	Supplementary Method 2	R
	Potential habitat suitability in PAs	Supplementary Method 3	R

The R code has been tested on R version 3.6.2 of Windows 10 oprating system

Supplementary Table 2

Kruskal-Wallis tests comparing the average richness of established alien animal species in 199,957 PAs between IUCN conservation categories, continents (Oceania, Europe, South America, Central America, Middle-east and Central Asia, South and East Asia, North America and Africa), 14 biomes, and island vs. mainland.

		Amphibian	Reptile	Bird	Mammal	Invertebrate	Overall
IUCN Conservation categories	$\chi2$	1166.7	492.64	9227.1	2324.5	967.17	7939.9
	P	<2.2e-16	<2.2e-16	<2.2e-16	<2.2e-16	<2.2e-16	<2.2e-16
Continent	$\chi2$	1159	491.63	7656.2	3581.5	937.08	4307
	P	<2.2e-16	<2.2e-16	<2.2e-16	<2.2e-16	<2.2e-16	<2.2e-16
Biomes	$\chi2$	931.75	2802.7	9393.8	2393.5	1432.4	6647.1
	P	<2.2e-16	<2.2e-16	<2.2e-16	<2.2e-16	<2.2e-16	<2.2e-16
Insularity	$\chi2$	508.56	132.99	149.8	1826.5	154.59	503.29
	P	<2.2e-16	<2.2e-16	<2.2e-16	<2.2e-16	<2.2e-16	<2.2e-16

Supplementary Method 1

R code used for network analyses to quantify the global flows of alien animals successfully established in PAs.

Abbreviations

flow.csv: file name. from: donor region, to: recipient region. num: number of introduction events.

```
library(dplyr)
library(circlize)
library(reshape2)

df = read.csv('flow.csv')

levels(df$from) =
c("Africa","Central_America","Europe","Mideast_and_Central_Asia","North_America","Oceani
a","South_America","South_and_East_Asia")
levels(df$to) =
c("Africa","Central_America","Europe","Mideast_and_Central_Asia","North_America","Oceani
a","South_America","South_and_East_Asia")

df = acast(df, from~to, value.var = 'num', sum)

#=====================PLOTTING THE CHORD
DIAGRAM==================================#
circos.clear()
circos.par(start.degree = 0,
        gap.degree = .6,
        track.margin = c(-0.1, 0.1),
        points.overflow.warning = FALSE)

grid.col = c(Africa = "#EE82EE", Central_America = "#66CDAA", Europe = "#8B7355",
Mideast_and_Central_Asia = "#FFEC8B",
          North_America = "#FFAEB9", Oceania = "#AB82FF", South_America = "#87CEFA",
South_and_East_Asia = "#FFA500")

chordDiagram(df, directional = 1,
          direction.type = c("arrows", "diffHeight"),
          transparency = 0.4,
          grid.col = grid.col,
          link.sort = TRUE,
          link.arr.width = 0.05,
          link.arr.type = 'big.arrow',
          link.lwd = .5,
          annotationTrack = c("grid", "axis"),
          link.border = 'darkgrey',
          ### scale = TRUE,
```

```
             self.link = 2,
             big.gap = 3,
             # order =
c("South_and_East_Asia","Mideast_and_Central_Asia","Central_America","South_America","
Oceania","Europe","Africa","North_America")
)

circos.trackPlotRegion(
  track.index = 1,
  bg.border = NA,
  panel.fun = function(x, y) {
    xlim = get.cell.meta.data("xlim")
    sector.index = get.cell.meta.data("sector.index")
    circos.text(
      x = mean(xlim),
      y = 2.9,
      labels = sector.index,
      facing = "bending",
      niceFacing = FALSE,
      cex = 0.7
    )
  }
)
```

Supplementary Method 2

Model formula for analysis of generalized mixed-effect model with a zero-inflated negative binomial distribution regression (ZINB).

<u>Abbreviations</u>

a: file name. alienr: alien richness in PAs, logarea: area of PAs (log transformed), logt: date of designation (log transformed). lognatr: richness of native species (log transformed), logf: human footprint in PAs (log transfomred), region: country identity of the PA.

```
zem <- glmmTMB(alienr ~ logarea+logt+lognatr+logf+(1|region), data = a, family = nbinom2)

# code of ZINB boxplot for Fig. 5 & Supplementary Fig. 4
library(ggplot2)
library(grid)
data <- read.csv("../zinb.csv",stringsAsFactors = FALSE)
data$all <- factor(data$all,levels = c("Native species richness","Designation year","Human
footprint","Surface area of PA"))

bplot_all <- ggplot(data,aes(y=all,x=Estimate))+
  scale_x_continuous(limits = c(-2,3.5))+
  geom_segment(aes(x=Estimate-2*SE,y=all,xend=Estimate,yend=all),size=0.6)+
  geom_segment(aes(x=Estimate+2*SE,y=all,xend=Estimate,yend=all),size=0.6)+
  #scale_color_manual(values = c("#006400","#708090","#8B2323","#00008B","red","green"))+
  geom_point(col="black", shape=0, size=2.6)+
  geom_point(aes(col=all), shape=15, size=2.6)+
  #scale_shape_manual(values = c(15,23,16,17))+
  scale_color_manual(values = c("#EE7621","#FFD700","#CD2626","#228B22"))+
  geom_vline(xintercept = 0, colour="red", linetype=2, size = .8)+
  xlab("")+ylab("")+theme_bw()+
  theme(legend.position='none')+
  theme(axis.text.y = element_blank(), panel.grid.minor = element_blank())

scale_x_continuous(limits = c(-0.8,2.5))+
geom_point(aes(col=all),size=2.6)+
axis.text.y = element_blank()

#######  scale_x_continuous(limits = c(-1,3)) ##########
#######  only rep and invert # scale_x_continuous(limits = c(-2.5,3.3)) ##########

vplayout <- function(x, y) viewport(layout.pos.row = x, layout.pos.col = y)
grid.newpage()
pushViewport(viewport(layout = grid.layout(2, 3)))
print(bplot_all, vp = vplayout(1, 1))
print(bplot_amp, vp = vplayout(1, 2))
print(bplot_rep, vp = vplayout(1, 3))
print(bplot_bird, vp = vplayout(2, 1))
print(bplot_mam, vp = vplayout(2, 2))
print(bplot_inver, vp = vplayout(2, 3))
```

Supplementary Method 3

R code for species distribution modeling to quantify habitat suitability of PAs for established alien animals.

Part a) sdm.modeling.projection.R code: this code is used to prepare for running the species distribution modeling (see the code file below (Part b), which is based on alien amphibian and can be used in any taxa)

```
# get a species coordinates (x and y) of the center of raster cells, a dataframe
# rast: a raster to define the research extent
# sp.df: species distribution points (real one)
 sp.grid.xy <- function(rast, sp.df, plotIf=F) {
        library(raster)
        library(rgdal)
   sp.df = data.matrix(sp.df)
        spatialSp<-SpatialPoints(sp.df,proj4string=CRS("+proj=longlat +datum=WGS84
+ellps=WGS84 +towgs84=0,0,0")) # caution on the projection
        index <- which(!is.na(getValues(rast)))
        rcells <- unique(cellFromXY(rast,spatialSp)) # species cell index
        rcells <- rcells[rcells %in% index]  # species cell index after removing NA
        if (plotIf) {
                ext <- extent(spatialSp)+c(-1,1,-1,1)
                plot(xyFromCell(rast,rcells),type="n",xlab="",ylab="",axes = F, frame.plot=F)
                plot(rast, ext=ext, add=T, legend=F)
                points(spatialSp,pch = 1,col="blue")
                points(xyFromCell(rast,rcells),pch = 16,cex=1.2)
        }
        return (xyFromCell(rast,rcells))
 }
##################################################################################
# species distribution models modelling
#sp_name: species name; vnames: the id of the combination (like clim, or comb); taxa: the class
of the species;

sdm.mp<-function(sp_name, vnames, taxa) {

cat('\n',sp_name,'modeling......\n')

library(biomod2)
library(PresenceAbsence)
library(ecospat)
perfor<-rep(9999,3)
names(perfor)<-c("accept_name","myRespName","num.point") # the result will be returned

myRespName <- paste(c(substr(unlist(strsplit(sp_name," ")),1,3),vnames),collapse="_") # the
output name from now on
perfor["myRespName"] <- myRespName
perfor["accept_name"] <- sp_name
```

```
# loading species occurrence
occ.sp1<-read.csv(paste("/home/sdmins10/sp_occ/",sp_name,".csv",sep=""),h=T)

# extract the grid cell coordinates where the species stand
occ.sp1<-as.data.frame(sp.grid.xy (bio[[1]], occ.sp1, plotIf=F))
occ.sp1[,"pa"]<-"a"

##################################################################################
# presence points in global environmental space
row.pa1<-merge(bioCoord, occ.sp1, by=c("x","y"),all = FALSE, all.x = T)
row.pa1<-row.pa1[order(row.pa1[,"id"]),]

# find rows where the species is present in global space
pa<-data.frame((!is.na(row.pa1[,ncol(row.pa1)]))*1)
names(pa)<-"pa" # create 1 column

# only a species has more than 15 grid cells will be used
if (sum(pa[,1])>=15) {

# assign NA to species global space for using background strategy
pa[which(pa[,1]==0),]=NA

 # presence background in environmental space
  if (taxa==" amphibia") back<-back_amphibia

  back<-as.data.frame(sp.grid.xy (bio[[1]], back, plotIf=F))
  back[,"PA"]<-"a"

# the background points in global space
  pbh<-merge(bioCoord, back, by=c("x","y"),all = FALSE, all.x = T)
  pbh<-pbh[order(pbh[,"id"]),]

  pbh<-data.frame((!is.na(pbh[,ncol(pbh)]))*1)
  pbh<-cbind(bioCoord[,1:2],pbh)

# use 30000 background points or all the points when the number is less than 30000
# add two repetition of pseudo-absences
# PA 1
  if ( sum(pbh[,3]) > 30000) {
		sam <- pbh[sample(which(pbh[,3]==1),30000),]

		bg<-merge(bioCoord, sam, by=c("x","y"),all = FALSE, all.x = T)
		bg<-bg[order(bg[,"id"]),]

```

```
        # for each PA repetition you have to take into account (coded by TRUE) ALL THREE,
presences, absences and pseudo-absences you want to work with (not only PA)
        bg<-merge(bg,occ.sp1, by=c("x","y"),all = FALSE, all.x = T)
        bg<-bg[order(bg[,"id"]),]
        bg[!(is.na(bg[,ncol(bg)])),ncol(bg)-1]<-1
        bg<-bg[,1:(ncol(bg)-1)]

        bg<-data.frame(as.logical((!is.na(bg[,ncol(bg)]))*1))
 } else {
                pbh[,"id"]<-bioCoord[,"id"]
                pbh<-merge(pbh,occ.sp1, by=c("x","y"),all = FALSE, all.x = T)
            pbh<-pbh[order(pbh[,"id"]),]
            pbh[!(is.na(pbh[,ncol(pbh)])),3]<-1
            pbh<-pbh[,1:3]

                bg<- data.frame(as.logical(pbh[,3]))}

 ### definition of data for the SDM
 myResp <- as.numeric(pa[,1])

 myRespCoord = bioCoord[,1:2]  ## coordinates of the all data
 myExpl<-stack(climRaster) # transform rasterbrick (crop from rasterstack) to rasterstack


 ### Initialisation
 myBiomodData <- BIOMOD_FormatingData(resp.var = myResp,
                 expl.var = myExpl,
                 resp.xy = myRespCoord,
                 resp.name = myRespName,

                                      PA.table = bg,
                                      PA.strategy =
"user.defined")

 # the real number of points will be used
 # perfor["num.point"]=sum(pa[,1])
 perfor["num.point"] <- sum(myBiomodData@data.species,na.rm=T)

 ### Options definition
 myBiomodOption <- BIOMOD_ModelingOptions()

 NbRun<-5 # Number of Evaluation run
 Model <- c('GAM','GBM','CTA','MARS','RF')
 NbModel <- length(Model)
 NM<-NbModel +2 # number of models plus two obs columns
```

```
  ### Modelling
  myBiomodModelOut <- BIOMOD_Modeling(
    myBiomodData,
    models = Model,
    models.options = myBiomodOption,
    NbRunEval=NbRun,
    DataSplit=70,
    Prevalence=0.5,
    VarImport=3,
    models.eval.meth = c('ROC','TSS'),
    SaveObj = TRUE,
    rescal.all.models = TRUE,
    do.full.models = FALSE,
    modeling.id = as.character(myRespName))

var_imp <- get_variables_importance(myBiomodModelOut) # relative importance of variable
write.csv(var_imp,file=paste(myRespName,"variable_importance.csv",sep="_"),row.names=T)

model.eva<-get_evaluations(myBiomodModelOut) #get model evaluations of calibrate data


# ensemble_modeling
myBiomodEM <- BIOMOD_EnsembleModeling(
                    modeling.output = myBiomodModelOut,
                    chosen.models = 'all',
                    em.by='all',
                    eval.metric = c('TSS','ROC'),
                    eval.metric.quality.threshold = c(0.6,0.8),
                                                models.eval.meth = c('TSS','ROC'),
                    prob.mean = T,
                    prob.cv = F,
                    prob.ci = F,
                    prob.ci.alpha = 0.05,
                    prob.median = T,
                    committee.averaging = F,
                    prob.mean.weight = T,
                    prob.mean.weight.decay = 'proportional' )

# get evaluation scores

write.csv(get_evaluations(myBiomodEM),file=paste(myRespName,"EM_evaluation
scores.csv",sep="_"),row.names=T)

###################################################
projection_curent
###################################################
```

```
# projection over the globe under current conditions
myBiomodProj <- BIOMOD_Projection(
                        modeling.output = myBiomodModelOut,
                        new.env = myExpl,
                        proj.name ='current',
                        selected.models = 'all',
                        binary.meth = 'TSS',
                        compress = 'xz',
                        clamping.mask = F,
                        output.format = '.grd')

# if you want to make custom plots, you can also get the projected map
# myCurrentProj <- get_predictions(myBiomodProj)

#####################################################
### EnsembleForecasting_current
#####################################################
myBiomodEF <- BIOMOD_EnsembleForecasting(
                     EM.output = myBiomodEM,
                     projection.output = myBiomodProj,
                                                    binary.meth ="TSS")

# reduce layer names for plotting convegences
# plot(myBiomodEF)


################################################################################

### save models evaluation scores and variables importance on hard drive

# nat_cal_eva<-
cbind(model.eva[1,1,,,],model.eva[2,1,,,],model.eva[2,3,,,]/100,model.eva[2,4,,,]/100,model.eva[
2,2,,,])
# colnames(nat_cal_eva)<-
paste(rep(c("auc","tss","sen","spe","cutoff_tss"),each=NbRun),"Run",c(1:NbRun),sep="_")

t_mo<-t(as.data.frame(model.eva))

write.csv(t_mo,file=paste(myRespName,"evalution.csv",sep="_"),row.names=T)

   }
   # RemoveProperly(myBiomodModelOut) #free both objects saved in memory and objects
saved on hard drive
return (perfor)
rm
(occ.sp1,row.pa1,pa,back,pbh,bg,bg1,myResp,myRespCoord,myExpl,myBiomodData,myBiomo
```

```
dModelOut,myBiomodEM,myBiomodProj,myBiomodEF,model.eva,var_imp,calib.lines,models.
prediction,cali_data_1,all_cali_data_1,mid,real_cali,tes,sam,Model,NM,NbModel,NbRun,myBio
modOption,myRespName,z,j,w,t_mo)
gc()
}
```

Part b) sdm.R code: this code is used to run the species distribution modeling, which is based on alien amphibian and can be used in any taxa

```
source("sdm.modeling.projection.R") # the code in Part a) above

library(biomod2)
library(snowfall)

# load the level and vnames
vnames<-"comb"
node<-1
ppn<-5
nodes<-node*ppn

# read species list
result <- read.csv("name_used.csv",h=T,stringsAsFactors=F) # just accept names

##################################################################################
########
# load climate, distribution data
# define the extent, following the raster "aq.tif"
e<-extent(-180, 180, -59.5, 83.67)

currentPath<-"cu10"  # the file path of environmental data at a resolution of 10 arcminutes

# current climate rasters in stack form


climRaster <- stack(paste0(currentPath,"/tav.tif"),
                    paste0(currentPath,"/pav.tif"),
                    paste0(currentPath,"/tsea.tif"),
                    paste0(currentPath,"/psea.tif"),
                    paste0(currentPath,"/tmin.tif"),
                    paste0(currentPath,"/tmax.tif"),
                    paste0(currentPath,"/pwet.tif"),
                    paste0(currentPath,"/pdry.tif"))
# tav: annual average temperature, pav: annual average precipitation, tsea: seasonal temperature,
psea: seasonal precipitation, tmin: the minimum temperate of the coldest month, tmax: the
highest temperature of the warmest month, pwet: the precipitation of the wettest quarters, pdry:
the precipitation of the driest quarters
```

```
# crop the raster
climRaster <- crop(climRaster, e)

# add 2 new rasters in another combination
if (vnames=="comb") {
# crop the rasters
ndvi<-raster(paste0(currentPath,"/ndvi.tif"))  # vegetation variable
ndvi <- crop(ndvi, e)

aq<-raster(paste0(currentPath,"/aq.tif"))  # water availability variable
aq <- crop(aq, e)
climRaster <- stack(climRaster,ndvi,aq) # stack the rasters
}

# load rasters to memory for slaves
#climRaster<-readAll(climRaster)

# baseline raster
# bio<-raster(paste0(currentPath,"/tav.tif"))
# bio <- crop(bio, e)
bio <- stack(climRaster) # it is a cropped rasterstack, to make sure all the cells have values

# extract the coordinates of land
bioCoord<-as.data.frame(coordinates(bio))
bioCoordValue<-extract(bio,bioCoord)
bioCoord<-cbind(bioCoord,bioCoordValue)
bioCoord<-na.omit(bioCoord)[,1:2]

# add id column for ordering the points
bioCoord[,"id"]<-c(1:nrow(bioCoord))

# load target background occurrence
back_amphibia<- read.csv("amphibia_10min.csv")

######################################################################
# parallel SDM
sfInit(parallel=TRUE, cpus=nodes)
sfLibrary(biomod2)

### Export data to nodes
sfExportAll()

sfClusterEval(ls())

# sdm.mp<-function(sp_name, vnames, taxa)
```

```
performance<-sfApply(result,1,function (li) return(sdm.mp(li[2],vnames, li[3])))

write.csv(performance,paste("performance_",vnames,".csv", sep=""),row.names=T)
sfStop()
rm(climRaster,bioCoord,bioCoordValue,bio)

gc()
}
```
