# Supplementary material for: Animal invaders threaten protected areas worldwide
Source: Nat Commun. 2020 Jun 8;11:2892. doi: 10.1038/s41467-020-16719-2 (PMC7280267; doi:10.1038/s41467-020-16719-2)
Supplement: Supplementary file 3 — Description of Additional Supplementary Files [file 41467_2020_16719_MOESM3_ESM.pdf]

Description of Additional Supplementary Files

File Name: Supplementary Data 1
Description: Databases and literatures used for collecting occurrence data of 894 alien animal species across the globe.

File Name: Supplementary Data 2
Description: GBIF sources and citations of species distribution data across taxa.
