# Supplementary material for: Animal invaders threaten protected areas worldwide
Source: Nat Commun. 2020 Jun 8;11:2892. doi: 10.1038/s41467-020-16719-2 (PMC7280267; doi:10.1038/s41467-020-16719-2)
Supplement: Supplementary file 6 — Reporting Summary [file 41467_2020_16719_MOESM6_ESM.pdf]

nature research

Corresponding author(s): Yiming Li
Last updated by author(s): May 15, 2020

Reporting Summary

Nature Research wishes to improve the reproducibility of the work that we publish. This form provides structure for consistency and transparency in reporting. For further information on Nature Research policies, see Authors & Referees and the Editorial Policy Checklist.

Statistics

For all statistical analyses, confirm that the following items are present in the figure legend, table legend, main text, or Methods section.

n/a	Confirmed	
☐	☒	The exact sample size (*n*) for each experimental group/condition, given as a discrete number and unit of measurement
☒	☐	A statement on whether measurements were taken from distinct samples or whether the same sample was measured repeatedly
☐	☒	The statistical test(s) used AND whether they are one- or two-sided<br>*Only common tests should be described solely by name; describe more complex techniques in the Methods section.*
☐	☒	A description of all covariates tested
☐	☒	A description of any assumptions or corrections, such as tests of normality and adjustment for multiple comparisons
☐	☒	A full description of the statistical parameters including central tendency (e.g. means) or other basic estimates (e.g. regression coefficient) AND variation (e.g. standard deviation) or associated estimates of uncertainty (e.g. confidence intervals)
☐	☒	For null hypothesis testing, the test statistic (e.g. *F*, *t*, *r*) with confidence intervals, effect sizes, degrees of freedom and *P* value noted<br>*Give P values as exact values whenever suitable.*
☒	☐	For Bayesian analysis, information on the choice of priors and Markov chain Monte Carlo settings
☒	☐	For hierarchical and complex designs, identification of the appropriate level for tests and full reporting of outcomes
☒	☐	Estimates of effect sizes (e.g. Cohen's *d*, Pearson's *r*), indicating how they were calculated

Our web collection on statistics for biologists contains articles on many of the points above.

Software and code

Policy information about availability of computer code

Data collection	No software was used to collect the data.
Data analysis	All statistical analyses have been conducted using the Circlize, glmmTMB and Biomod2 packages with the version 3.6.2 of the R software and the ESRI ArcGIS 10.2.1. All the codes used are available in the Supplementary Method 1-3.

For manuscripts utilizing custom algorithms or software that are central to the research but not yet described in published literature, software must be made available to editors/reviewers. We strongly encourage code deposition in a community repository (e.g. GitHub). See the Nature Research guidelines for submitting code & software for further information.

Data

Policy information about availability of data

All manuscripts must include a data availability statement. This statement should provide the following information, where applicable:

- Accession codes, unique identifiers, or web links for publicly available datasets
- A list of figures that have associated raw data
- A description of any restrictions on data availability

The study species list is based on the IUCN (www.IUCN.org), Kraus' (2009) compendium (https://link.springer.com/chapter/10.1007/978-1-4020-8946-6_6), Capinha et al. (2017) (http://dx.doi.org/10.1111/ddi.12617), Liu et al. (2019) (https://doi.org/10.1016/j.cub.2018.12.036), the BirdLife International & NatureServe geodatabase (http://datazone.birdlife.org/species/requestdis), the Global Avian Invasions Atlas (http://dx.doi.org/10.6084/m9.figshare.4234850), Long's (2009) book (https://ebooks.publish.csiro.au/content/introduced-mammals-world), Capellini et al. (2015) (http://dx.doi.org/10.1111/ele.12493), and Global Invasive Species Database (http://www.iucngisd.org/gisd/). All occurrence data used in this paper are freely available from the web databases including GBIF (http://www.gbif.org/), ALA (http://www.ala.org.au/), Arctos (http://arctos.database.museum/), CBIF (http://www.cbif.gc.ca/), EUNIS (http://eunis.eea.europa.eu/), HerpNET (the original website was taken down, now replaced with the VertNet portal http://www.vertnet.org/search), iNaturalist (http://www.inaturalist.org/), NMNH (https://naturalhistory.si.edu/research/vertebrate-zoology), NCMNS (http://collections.naturalsciences.org), SpeciesLink (http://www.splink.org.br/), USGS Nonindigenous Aquatic Species Database (http://nas.er.usgs.gov/), Vertnet (http://portal.vertnet.org/), ORNIS (http://ornis2.ornisnet.org/), Global Avian Invasions

Atlas (http://dx.doi.org/10.6084/m9.figshare.4234850), BirdLife International & NatureServe (http://datazone.birdlife.org/species/requestdis) and 959 published references (Supplementary Data 1-2). We obtained data on the location, boundary, designation year, and area of global protected areas from the April 2019 version of the World Database on Protected Areas (www.protectedplanet.net). Human footprint data are available from the Last of the Wild Project website (https://sedac.ciesin.columbia.edu/). Native amphibian, bird and mammal richness data are available on from Biodiversity Mapping website (https://biodiversitymapping.org/wordpress/index.php/home/). Reptile native richness is calculated based on distributional range maps extracted from Dryad (https://doi.org/10.5061/dryad.83s7k). All climatic data are available on the WorldClim-Global Climate Database (www.worldclim.org). Vegetation data are available on the Nasa Earth Observations (https://neo.sci.gsfc.nasa.gov/). Water data are available on the global lakes and wetlands database (GLWD, https://www.worldwildlife.org/pages/global-lakes-and-wetlands-database).

Field-specific reporting

Please select the one below that is the best fit for your research. If you are not sure, read the appropriate sections before making your selection.

☐ Life sciences ☐ Behavioural & social sciences ☒ Ecological, evolutionary & environmental sciences

For a reference copy of the document with all sections, see nature.com/documents/nr-reporting-summary-flat.pdf

Ecological, evolutionary & environmental sciences study design

All studies must disclose on these points even when the disclosure is negative.

Study description	Protected areas play a unique and crucial role in conserving our global biodiversity. While protected status has the potential to affect human impacts within these areas, one of the increasing and major threats to global biodiversity is invasive alien species, which do not care about the legal designation of an area. However, the degree to which alien species have established across global protected areas and their potential drivers remain unknown. Our present study aims to identify those protected areas harboring high richness of alien animals (including vertebrates and invertebrates) and their potential drivers, which may provide timely and important information to develop efficient prevention, detection, and rapid response strategies against new invasions in protected areas worldwide.
Research sample	We compiled a comprehensive distribution database comprising spatially-explicitly occurrence records of 894 established terrestrial alien animal species across 11 taxonomic groups from GBIF, ALA, Arctos, CBIF, EUNIS, HerpNET, iNaturalist, NMNH, NCMNS, SpeciesLink, USGS, Vertnet, ORNIS, GAIA, BLINS and 959 published references in Supplementary Data 1-2 for 199,957 global protected areas from the April 2019 version of the World Database on Protected Areas (WDPA). The web link for these databases are available as noted above.
Sampling strategy	We identified the species used and collected occurrence data for each species based on careful collections from various widely used databases on global alien animal establishments, and an intensive review of published references which are both provided in supporting data 1-2.
Data collection	X. Liu, X. Wang, T. Song, C. Huang and Y. Li collected the data from the databases and references as noted the above.
Timing and spatial scale	We collected the alien animal occurrence data from January, 2014 to October, 2019. We obtained native and alien range information for alien animals from the IUCN database on January, 2018. We obtained data on the global protected areas from the World Database on Protected Areas (WDPA) on April 2019. Our study was conducted at the global scale.
Data exclusions	We only included protected areas with detailed geographical information and a status of “designated” (not including those designated as UNESCO Man and Biosphere Reserves), “inscribed”, or “established” for data analyses. We followed WDPA best practice guidelines and dissolved the overlapped areas into a single polygon, assigning overlapping areas as the stricter IUCN conservation category. We removed those species re-introduced into a species’ native range, released within their native ranges, experimentally introduced into small islets, and that represented questionable introductions without robust evidence. We also removed those species that have not established feral populations, or species whose alien ranges are unclear or without precise geographical location data. The exclusion criteria were based on previous global studies on protected areas (e.g., Jones et al. 2018, 10.1126/science.aap9565).
Reproducibility	To explore the robustness of our analyses, we conducted a series of sensitivity analyses to ensure reproducibility. All data sources have been provided in the supporting materials.
Randomization	This is not relevant for our study. We used a random factor (region identity) to control for the possible spatial pseudo-replication.
Blinding	Blinding was not relevant with our study because all data used in the present study was collected from public databases and published references, which have been provided in the supporting material.

Did the study involve field work? ☐ Yes ☒ No

Reporting for specific materials, systems and methods

We require information from authors about some types of materials, experimental systems and methods used in many studies. Here, indicate whether each material, system or method listed is relevant to your study. If you are not sure if a list item applies to your research, read the appropriate section before selecting a response.

Materials & experimental systems

n/a	Involved in the study
☒	☐ Antibodies
☒	☐ Eukaryotic cell lines
☒	☐ Palaeontology
☒	☐ Animals and other organisms
☒	☐ Human research participants
☒	☐ Clinical data

Methods

n/a	Involved in the study
☒	☐ ChIP-seq
☒	☐ Flow cytometry
☒	☐ MRI-based neuroimaging
